# Family Recovery Interventions with Families of Mental Health Service Users: A Systematic Review of the Literature

**DOI:** 10.3390/ijerph18157858

**Published:** 2021-07-25

**Authors:** Michael John Norton, Kerry Cuskelly

**Affiliations:** 1 National Engagement & Recovery Lead, Mental Health Engagement & Recovery, St. Loman’s Hospital, Palmerstown, 20 Dublin, Ireland; 2 Adult Continuing Education, The Laurels, University College Cork, College Road, T12 YN60 Cork, Ireland; 3 Principal Social Worker, Adult Mental Health Services, North Dublin, Ireland; kerry.cuskelly@gmail.com

**Keywords:** family, recovery, mental health, interventions, enablers

## Abstract

Introduction: Recovery has become a catalyst for much organisational and cultural change within mental health services. Recovery involves the service user living the best life of their choice despite the presence of mental health challenges. In contrast, recovery of families remains under-developed with minimal attention given to the unique support families may require in their own recovery journeys. This paper aims to place focus on the topic through a systematic review of the literature into family recovery interventions in mental health; Method and Analysis: A PRISMA compliant systematic review was initiated. It included how the reviewers retrieved and selected studies for the systematic review. It outlined the inclusion/exclusion criteria and how these were further developed through the PICO framework. It also outlined how the reviewers assessed issues of bias and quality, as well as the process of data synthesis; Results: Three studies were included in this review. Each focusing on family recovery interventions across the lifespan: Kidstime to family toolkits to family psychoeducation. The benefits and challenges of each intervention to the family were synthesised along with a list of four family recovery enablers that are vital for the implementation of such family recovery interventions; Discussion/Implications for Practice: The results highlight the paucity of quality literature available for family recovery interventions. All three studies scored poorly in terms of quality, with one particular study (Nagi and Davies 2015) lacking quotations from participants to back up their claims. From this study, a number of actions need to be implemented, specifically around the enablers needed to allow for family recovery interventions to be fully implemented.

## 1. Introduction

In recent years, mental health services in Ireland have adopted a recovery-orientated paradigm/model [1]. The concept of recovery within mental health ranges from clinical recovery [2], to self-defined personal recovery and lately to the idea of social recovery [3]. A commonly used definition of personal recovery within mental health originates from the scholarly work of William Anthony, who states that recovery is:

“…a deeply personal, unique process of changing one’s attitudes, values, feelings, goals, skills, and/or roles. It is a way of living a satisfying, hopeful, and contributing life even within the limitations caused by illness. Recovery involves the development of new meaning and purpose in one’s life as one grows beyond the catastrophic effects of mental illness” [4] (p. 21).

The notion of personal recovery as a journey, that focuses on quality of life and meaning beyond a broader set of psychosocial parameters is distinct to the idea of clinical recovery which focuses more on examining psychopathology, symptom alleviation and functionality among others [5]. These two central notions are not mutually exclusive, with the recovery agenda in mental health services offering space for multiple ideologies and perspectives to co-exist. National initiatives such as Advancing Recovery in Ireland (ARI) [6], Clinical Programmes in Mental Health [7], Enhancing Teamwork [8] and recovery educational programmes such as *Eolas* [9], developing under these auspices. It is important that policies developed in the area of mental health, therefore, reflect the lived experience of mental health and the concept of recovery as understood by those who use and interact with mental health services at both an individual and familial level.

### 1.1. Context to Family Recovery in Ireland

“*Sharing the Vision*” [10] is the current roadmap for mental health service provision in Ireland. As part of its recommendations, “*Sharing the Vision*” advises that peers, i.e., service users and their families, should be core to the development and running of mental health services [10]. Since the inception of “*A Vision for Change*” [11], the precursor to “*Sharing the Vision*”, many documents have been cited and built on this recommendation [12,13,14].

### 1.2. Importance of Inclusion of Families

At least 182,884 people identify as family carers in Ireland [15]. While family carers tend typically to identify as female [16], family carers in mental health can also identify as male, as parents, as siblings, children and non-relatives, for example, close friends or supporters. National policy in Ireland acknowledges the need to support family carers [17]. The issue of family burden within mental health is an internationally recognised phenomenon [18,19], with evidence-based tools developed to specifically measure this [20,21,22]. International research focusing on families in mental health has also examined the quality of life [23,24], understanding a loved one’s mental illness [25,26], and the impact of caregiving on the carer themselves [27,28]. Alongside the growing research base, countries across the globe have translated this research into practice by legislating for the provision of supports for carers, for example, “*The Care Act 2014*” (England) [29], offering a legislative basis for providing a needs assessment for carers. In Australia, “*The Carer Recognition Act*” [30], states that carers should be treated with respect and considered as partners with other care providers. In the Republic of Ireland and in Northern Ireland there is a legislative basis for the provision of financial support for carers within “*The Social Welfare (Carer’s Allowance) Regulations*” [31] and “*The Carers and Direct Payments Act*” (Northern Ireland) [32], respectively. There is also national legislation in the Republic of Ireland that provides financial support for carers if they need to take leave from work due to their caring responsibilities under “*The Carers Leave Act*” [33].

A modern and progressive mental health service, as outlined in *Sharing the Vision*, should offer a range of support options to families as well as service users from psychoeducation to group-based aides and peer support. Amongst these services fits’ recovery interventions for families. It is with this in mind that this review was carried out.

### 1.3. Rationale for Systematic Review

This review was required for a number of reasons. Firstly, while it was acknowledged in both national [12,13,14,17,34,35], and international contexts [36,37,38,39], that offering support to families linked with mental health services is important, the authors could find no available synthesis of evidence to recommend what recovery interventions exist for families in mental health services in their own right. It is therefore suggested that an evidence base was needed in such areas to guide best practice. This knowledge would benefit service users and service providers as it is recognised that supporting families has positive effects in relational [40] and economic contexts [41]. Secondly, within the context of delivering on specific targets set out by the Health Service Executive under the auspices of “*A National Framework for Recovery in Mental Health*” [42], the “*Family Recovery Guidance Document 2018–2020*” [35] outlines a comprehensive approach to implementing recovery approaches for families in mental health services in Ireland. The guidance articulates that, “*In families where a member has a mental health issue, the family also require support for their own recovery*”, [35] (p. 10).

## 2. Material and Methods

As such, a systematic review of the available literature was conducted to address the following objectives:

To identify what recovery interventions exist for families of loved ones attending mental health services that focus on the family member in their own right.

To identify key enablers of family recovery interventions.

The review was compliant with the Preferred Reporting Items for Systematic Reviews and Meta-Analyses (PRISMA) standardised reporting guidelines [43] PRISMA is an approach developed by multiple stakeholders which provides guidance on how best to conduct and report systematic reviews and meta-analyses so that their methods can be reproducible and transparent.

### 2.1. Protocol and Registration

It is now recognised as best practice when conducting a systematic review, to also create a protocol. This is useful for several reasons including (1) avoiding duplication of work, (2) maintaining accountability and (3) maintaining the rigor associated with systematic reviews. All protocols for systematic reviews are registered with Cochrane once published and a serial number assigned. The reviewers created a protocol for this systematic review. However, we were unsuccessful in registering it. As such, the protocol for this systematic review was not published or registered with Cochrane.

### 2.2. Eligibility Criteria

Initially, a generic inclusion/exclusion criterion (Table 1) was developed to support the reviewers in selecting relevant articles. However, to support the reviewers in being as thorough as possible, an additional inclusion/exclusion criteria table was also created based on the principles of PICO [44] (Appendix A). PICO is useful in creating research questions as it breaks down the question into four essential components: population, interventions, comparisons and outcomes [45]. See Appendix B for the detailed PICO explanation sheet for this review.

### 2.3. Search Strategy

A primary search was undertaken using ten databases: CINAHL, JSTOR, OVID SP, PsycARTICLES, PsycINFO, PubMed, Science Direct, Web of Science, Wiley Online Library and EBSCHO host. Following this, secondary searches were undertaken using repositories and sources such as ResearchGate. Finally, to strengthen the review process further, a third round of searching was employed using the references from already included papers (Appendix C).

A search time limit of 10 years (2010–2020) was adhered to by both reviewers to ensure that only the most up-to-date, best-available evidence was collected. The search terms used included: “family”, “supporter”, “spouse”, “partner”, “significant other”, “caregiver”, “relative”, “sibling”, “parent”, “adult”, “mental”, “psychiatric”, “mental illness”, “psychiatric disorder”, “mental ill health”, “intervention”, “group work”, “therapy”, “education”, “support”, “recovery”, “wellbeing”, “self-care”, “burden” and “quality of life”.

### 2.4. Study Selection and Data Extraction

#### 2.4.1. Screening

There were three rounds of screening employed for this systematic review. Round one encompassed a review of titles from the search results. When reviewing titles, the reviewers saved any documents relating to family recovery into a round one folder. This folder was further divided to show documents that were found through databases and those found within repositories and other sources. Round two involved reading abstracts/executive summaries of all articles saved in the round one folder. During this time, duplicates were removed and saved in the duplicate folder within the round two folder. The initial inclusion/exclusion criteria (Table 1) were applied to the remaining documents and any document not relating to the research question was excluded. In the final screening round, the full text of all remaining articles was reviewed and read to ensure they all met the in-depth inclusion/exclusion criteria highlighted in Appendix A. Once this screening process was completed, the remaining articles were included in the review.

#### 2.4.2. Data Collection and Extraction

A data extraction form was developed. Study data were extracted and placed in a Microsoft Word document for purpose of data collation. This was completed by both reviewers to ensure accuracy. Disagreements between authors were resolved through the re-analysis of the particular paper in question, along with a discussion on the next steps. A trial of the data extraction form was conducted on the first two studies that met the inclusion/exclusion criteria to ensure that it captured all aspects and intricacies of the findings. Data extracted from studies included: authors, year, geographical location, study aim, sample, sample size, age range, setting, methodological approach and theoretical orientation of the given study.

#### 2.4.3. Assessing the Risk of Bias

As part of this review, bias was assessed by both reviewers (MN, KC) as part of the quality appraisal process. During the assessment, the reviewers assessed bias under the headings: *selection bias*, *performance bias* and *attrition bias*. Disagreements between reviewers were resolved by consensus.

#### 2.4.4. Assessing the Quality of Evidence

An adaptation of [46] critical appraisal tool was used to assess study quality. The tool was originally comprised of nine questions in which answers were rated from good to very poor. However, the [47] adaptation converts these ratings into numerical values resulting in a score that measures study quality. Each study can receive a minimum of nine up to a maximum of 36 points allowing for study quality to be graded [47]. This tool allowed the reviewers to validate included papers as it initiated a process whereby the reviewers could reflect on these studies systematically.

#### 2.4.5. Data Analysis

The reviewers adopted a process of thematic analyses [48] to analyse and synthesise the data. This involved the reviewers becoming familiar with the data through many readings of included papers. The initial codes were generated and discussed resulting in themes. These were revised, adjusted and refined by both reviewers. The process was supported by critical engagement from both reviewers with their own subjectivities and pre-conceived ideas about the research question under inquiry and this was further supported through reflections.

## 3. Results

**Figure 1:**
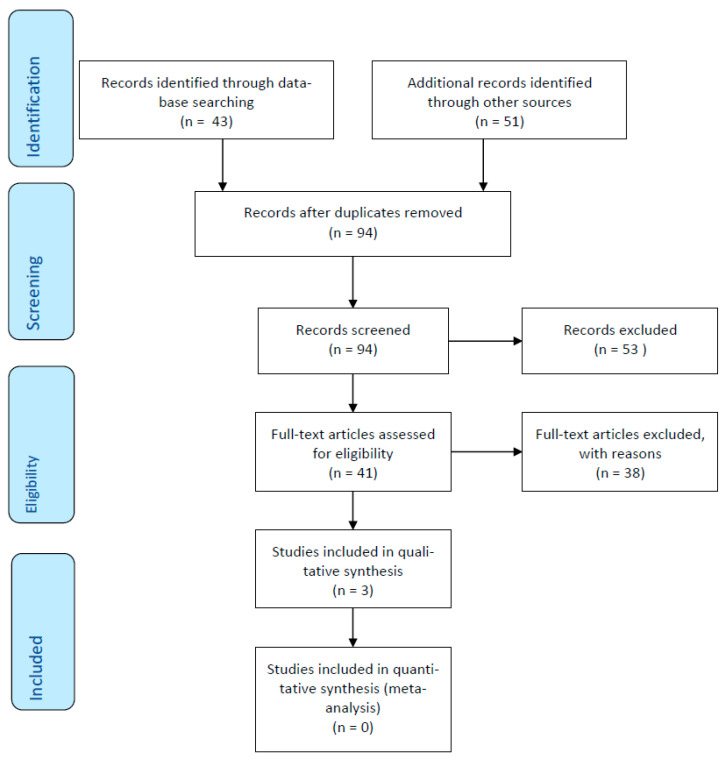
PRISMA Flow Chart.

Initially, there were 94 hits that specifically related to family recovery interventions within mental health services. This was narrowed down to 41 hits based on the research question. Further restrictions were imposed by the inclusion/exclusion criteria. After implementing the more detailed inclusion/exclusion criteria (Appendix A), Three studies remained which described family recovery interventions in mental health settings (Figure 1). Only qualitative studies were included in this review to coincide with our review aims, particularly aim two, identifying key enablers of family recovery interventions. Added to this, meta syntheses were also excluded as their results are not necessarily true reflections of the papers reviewed as reviewers are two times removed from the original study data. As such, meta-syntheses are interpretations of previous findings which may not reflect the actual findings of reviewed papers.

The studies consisted of three research papers, all of which employed a variety of qualitative methodologies, service settings and category of family members. Two studies included parents within their sample with one paper respectively including partners [49] and siblings [50]. Only one study included the perspectives of children [51]. Two studies were set in the community, whereas one study was based within a low-security forensic mental health facility. Two studies provided an age range for participants with none distinguishing participants as male, female or other. No studies described their methodological or theoretical orientation. Further detail can be found in Table 2. Table 3 details the results of a quality appraisal process with Table 4 providing a synopsis of included studies. Finally, Table 5 describes the emerging themes and sub-themes from included studies, which are now presented.

### 3.1. Family Recovery Initiatives

From the included studies, three family recovery interventions were identified. These included a family recovery toolkit [49], family psychoeducation [50] and Kidstime [51]. Two interventions were based within a non-NHS community setting [49,51]. The family psychoeducation intervention, in this instance, was delivered within a low security forensic mental health setting. Interventions covered family members across the lifespan, from children [51] to parents [49,50] to partners and other relatives [49].

### 3.2. Benefits

Included studies mentioned several benefits to the aforementioned family recovery interventions. These included: education, social inclusion and facilitation of discussions on difficult topics. Such benefits are now discussed.

#### 3.2.1. Education

Two out of the three interventions discussed within this review have educational components to their respective intervention [49,50]. Ref. [49] discuss the creation of a toolkit specifically for families of those with mental health difficulties. This resource was highlighted by participants as highly valuable due to the “*trusted*” information provided in it by service providers [49]. Such information was presented in various formats including literature-based, auditory and visually-based through books, CDs and DVDs.

Like [49], ref. [50] reported similar findings in terms of the value ability of the information they received. This time through the use of family psychoeducation [50]. Participants here suggested that they also found the information useful, with material that was provided, presented in such a format that families found accessible and user-friendly [50]. Ref. [49] add to this suggesting that although it is important to have material that is accessible and user-friendly, it needs to have the necessary information vital for families to support themselves and their loved ones experiencing such difficulties. Such information includes: (1) differing mental health challenges and their treatments, (2) structure and function of the mental health services, (3) support during crisis, (4) legal rights and financial issues resulting from mental ill-health, (5) techniques to address and talk about difficulties, (6) techniques to manage aggression and violence, (7) early warning signs and signs of relapse, (8) accessing necessary supports, (9) managing carer stress and (10) family member narratives [49]. The interest in learning other aspects of mental health and how it links to various acts and behaviours was also identified by [50] where their participants also learned and understood the link between mental ill-health and offending behaviours.

Despite the lack of educational interventions associated with the [51] study. Education was also a major feature for those interviewed as part of their study on the effects of Kidstime on family recovery. Here, it was noted that kids valued the opportunity to learn about their parent’s mental ill-health through a variety of means, including gaming [51].

“I’d say, this is a bit silly but I like, I quite like the games and like the easier way of understanding it.” [51]

This increased knowledge allowed the children involved to speak more freely with their understanding peers regarding their familial situation resulting from parental mental ill-health and their fears of becoming mentally unwell themselves [51].

“When I started the Kidstime project, I felt like I couldn’t really express myself, because I know that people often thought that because my mum had mental illness I may have mental illness, so I didn’t want to say anything, because I didn’t want to seem odd or say anything inappropriate, so I kept to myself. So when I started coming to this project, you realize that, not necessarily, because when you know that other people have the same problems as you, and they look normal, they seem normal, that’s its okay to come out and just, you know, express yourself a bit more. So I just felt like I wouldn’t necessarily, after learning about the illness I felt like I wouldn’t necessarily become mentally ill, so it’s okay for me to express myself.” [51]

#### 3.2.2. Social Inclusion

Only one study described a recovery intervention that surrounded a social activity with others within the mental health community: Kidstime [51]. This social recovery intervention is specifically tailored for children of parents that have a mental health challenge. The club has several benefits including decreasing isolation felt by such children through social inclusion in group activities. Through such inclusion, these young people are provided with space and opportunity to play and have fun, like other kids their age [51].

“We play games and we just talk about life and then if someone has an idea and then we talk about that.” [51]

According to [51], having this opportunity to be children also has the added benefit of reducing familial strain caused by the mix-up of roles experienced within the family unit due to parental mental ill-health.

#### 3.2.3. Facilitation of Discussions of Difficult Topics

Two studies in this review discussed how their representative initiative (recovery toolkit and Kidstime) acted as catalysts for these family members to discuss difficult topics [49,51]. According to [49], the developed toolkit provided the family with tools and techniques to discuss difficult topics, including hearing voices and delusional beliefs. It also provided signposting information for such individuals to join peer support groups where they could discuss such difficult topics with others who have experienced similar difficulties within their caring role [50]. Ref. [51] also identified such benefits within the running of Kidstime, where they found children attending the service learned the ability to share any issues they may have with their peers. Kidstime also allowed the opportunity for facilitated discussions with this cohort on topics that are not openly discussed elsewhere, which has proven beneficial for the children involved [51].

“Home was sad, Kidstime was fun. That’s what I looked forward to. I looked forward to having fun, you know being a child. But at home you have to be an adult, look after yourself, look after mum, look after the house, give her medication; at Kidstime you’re having fun. You’re being looked after and you’re not looking after others…. there are people there who are paying attention to you and you can go and speak to because you probably can’t speak to your mum because you know she’s not well she probably won’t understand. But Kidstime was time for the kids; I think that’s why it’s called Kidstime.” [51]

### 3.3. Challenges

The above family recovery interventions are also affiliated with several challenges that impacts on not just the service but those participating in such interventions Such challenges were themed and include: hidden interventions, practicalities and age-appropriate interventions. These are now presented.

#### 3.3.1. Hidden Interventions

Two out of the three included studies cited hidden interventions as a challenge to such family recovery interventions in mental health [49,50]. According to [50], participants never received information on family psychoeducation before receiving such interventions. This is expanded upon in the second study where participants found it difficult to access mental health services and when they finally were granted access, they discovered interventions that would have benefitted if there was access to it at first contact [49]. This alarmed participants in the [49] study as they had concerns regarding the availability of the recovery toolkit when needed. This can be related to the continuous presence and primacy of the biomedical model as highlighted by [50].

#### 3.3.2. Practicalities

Two of the included studies highlighted practicalities as a potential challenge to implementing such family recovery interventions [49,51]. According to [49], such practicalities relate to access and support. In terms of access, participants discussed the need for such a toolkit to be available 24 h a day [49]. However, this provided practical challenges such as the ability of staff to support family members in navigating the content of the toolkit [49]. Within [51] study, such practicalities related to attending the Kidstime programme and included transport and group size. Group size, in particular, can cause challenges to members participants are more likely to present as shy or worried about the unknown, all of which can cause the child to become overwhelmed and further increase vulnerability, thus lessoning the positive effects Kidstime has on individual participants [51].

#### 3.3.3. Age-Appropriate Interventions

Ref. [51] was the only study included that discussed interventions for children of parents with a mental health challenge. Within their analysis of the effects of Kidstime, they identified that although children of all ages and abilities had fun, older children raised the need for activities to be age-appropriate in order to suit the older child cohort that attends the service.

“I would like more people my age around.” [51]

“The only downside, what I was going to say before but I didn’t want to say anything, was that, I felt more attention was paid to the younger kids than to the 15, 16 year olds.” [51]

### 3.4. Enablers of Family Recovery Interventions

The three studies included in the review also identified several enablers of family recovery interventions based on their research into the three family recovery interventions. Such enablers surround the inclusion of written information, access, supports and finally how decisions are made to attend/take part in interventions discussed above. These are now discussed.

#### 3.4.1. Written Information

One of the included papers cited the use of written information as a way to support family recovery moving forward [49]. Within their study, participants identified that the lack of written information had impacted their family’s recovery journey as this led them to seek information from less reliable routes, such as the internet [49]. This, according to [49] led to such participants feeling overwhelmed by the amount of information available. Thus, the toolkit developed as part of the [49] study was perceived as highly valuable to participants as the toolkit provided information and signposting which came from a “*trusted*” source [49]. The importance of such information is highlighted in the following quotation:

“……even just pointers to that, where you can go on and ask questions because a lot of people have, like I say great knowledge of how to deal with things and it may not be the right ones for you but it it’s ideas isn’t it, to trigger you.” [49]

#### 3.4.2. Access

Participants from all three studies reviewed all mentioned, to some degree, the issue of access either to information [49,50] or to a community group [51]. From the two studies that discussed access to information, ref. [49] participants highlighted how they received no reliable information about their family member’s diagnosis or how such participants can support their loved one through their difficulties until the toolkit was created.

“I think maybe, that if there had have been a pack when I was going through it…., maybe the outcome for my son would have been different than it is now.” [49]

However, unlike the previous study, ref. [50] participants did receive information, but only after the said intervention had occurred. Unlike the previous studies, [51] described access in terms of travel to their intervention. Here, participants identified the difficulty in getting appropriate travel to the intervention (Kidstime) as a barrier to the overall evaluation of Kidstime [51].

#### 3.4.3. Support

Only one study had recommendations regarding support [49]. Although participants within this study were in favour of such a toolkit, they also feared that such interventions could be used to reduce face-to-face contact with service providers [49]. Additionally, ref. [49] made a recommendation regarding the toolkit itself. Here, participants were also concerned that the toolkit was not totally focused on the family of the person with a mental health challenge, instead, some material related to the service user themselves in the hope that this would indirectly support the relatives [49].

#### 3.4.4. Decision Makers for Attending Interventions

Only one of the studies reviewed [51] discussed the factors or lack of identified factors that influence participants to attend a family recovery intervention. Here, ref. [51] highlighted that for the intervention they were evaluating (Kidstime), they could not identify the various decision-makers (factors that determine attendance) for those attending the intervention.

“I felt shy, I wondered what it was going to do, what it was about. Now I know. It is about having fun and mental illness so when my mum or dad get ill, you can help them.” [51]

## 4. Discussion

There was evidence found in the review to suggest that family recovery interventions have multiple positive effects for families however, these are off-set by perceived barriers to being able to access such supports in terms of gate-keeping of information by service providers, the necessity of the service provider’s input in terms of supporting people through the recovery intervention and age-appropriateness of these interventions. Within the review findings, key enablers were identified that address such barriers and more. These enablers, if implemented correctly will allow all categories of family members to feel more involved in the entire recovery process. Findings from the review suggest that recovery interventions can take many forms but at a basic level should include offering consistent, reliable written information to families, ensuring information provided is accessible at all stages of the recovery journey particularly at the beginning and that recovery interventions are not conditional, i.e., that provision of recovery interventions should not take away from face-to-face interactions with service providers.

### 4.1. Results in the Context of the Current Literature

To the author’s knowledge, no previous reviews have been carried out focusing on family recovery interventions [52]. The findings from this review demonstrate that family interventions can have multiple benefits but face challenges to implementation due to barriers at the service provider level that are consistent with another review recently carried out [53]. Both Department of Health and HSE strategy [17,35] refers to the need to offer families information and support in their own right, subjects which concur with the findings from the review in the context of what families find beneficial to aid their own recovery journey. Previous individual studies have noted the benefits of psychoeducation [9,26], peer support [13,35] and having input from mental health practitioners in terms of support navigating difficult conversations [18,27].

Families consistently articulate a need for written information [9,13] and for access to information at the right time [13]. Families also articulate a wish for support from input from qualified mental health practitioners in their own right as family members [13].

Less clear are articulations of reasons why family members do or do not engage in interventions. While this was found to be one of the potential enablers of recovery from this review, it is not possible to extrapolate this as a definite enabler of family recovery.

### 4.2. Strengths and Weaknesses of the Review

This review is possibly the first to examine the topic of family recovery interventions under the specific parameters set out at the beginning of the review. We used robust methods to identify, select, appraise and synthesise the articles included in the review. We followed accepted standardised reporting guidelines to ensure rigour in the reporting of the research. The review included articles on people identifying as families across the lifespan, of adults with mental health difficulties.

The findings of the review are limited in the generalisable conclusions that can be drawn due to the poor quality of the included appraised articles across all domains in our chosen critical appraisal tool. The articles included in the review are all drawn from the UK which limits generalisability across different mental health settings globally. The [50] article did not include quotations to back up their findings which limited our ability to assess their article fully. The [49] article had some quotations but very little related to their findings. The only paper that had quotations to back up evidence was [51]. Thus, as a result, the quotations used in this review mainly come from [51] which may cause overdue attention to this article. However, in the reviewer’s defence, this resulted from poor reporting mechanisms employed by both [49,50]. 

### 4.3. Risk of Bias

The risk of bias was accessed using an adapted version of a protocol developed by [46] and is presented in Table 3. This tool was chosen as it was developed specifically to appraise the quality, including the risk of bias, of disparate studies [46] (p. 1292) and to combat the difficulty found traditionally in assessing qualitative research. This is due to a tendency towards lack of reporting of criteria such as validity, reliability and bias [53] which can in turn make it difficult to critically appraise studies in a systematic way.

### 4.4. Implications for Practitioners and Areas for Further Research

The findings of the review assist to bridge a gap in the literature and provide evidence on the types of interventions available to families in mental health services. It also highlights gaps that may exist in the provision of services to families in these settings. This information is important not only for future research on understanding family’s needs in the recovery process but also for identifying the types of recovery interventions mental health practitioners should be developing and facilitating in order to ensure the provision of high-quality, effective services to families in mental health settings.

Understanding what families need to support their own recovery journey as distinct from the recovery journey of their loved one in mental health settings and knowing how to put this into practice, is a necessary aim for mental health practitioners. The findings from this review could assist mental health practitioners to address practical gaps in service provision for families within their services using an evidence-informed approach.

## 5. Conclusions

Practical applications of this review, such as ensuring families are given appropriate information at an early stage of their recovery journey, should be easily addressed and acted upon. Added to this, the fact that national policy in Ireland supports the views put forward by families in this systematic review should ensure that mental health practitioners feel empowered to activate these changes within their workplace settings.

As articulated by families themselves, there are a number of areas that could benefit from more in-depth research to assist in strengthening the evidence-base for family interventions and their constructs. This includes looking at why information may be gate-kept from families which could otherwise greatly assist them in navigating their recovery journey and that of their loved ones, what investment is needed from mental health services to adequately support families in their recovery journeys and what the unique aspect of recovery interventions for distinct family groups are, e.g., young family members, adult family members, LQBTQI+ family members and so on.

With the journey towards recovery-focused mental health services for service users fully underway and becoming embedded in organisational and cultural ways across services, it is now incumbent on services to invest similarly in supporting families in their recovery journeys.

### Final Reflections

The results of this review mirror much of the cultural and organisational attitudes towards families of those with mental health challenges. The results are created from few and poorly reported studies which imply a paucity of attention given to family recovery. As such, as service moves forward and develops, one must be cognisant that the family has their own recovery journey to follow too. Added to this, the process of recovery for families is somewhat similar to that of the service user, albeit with some key distinctions. This is most clearly evident through the enablers of family recovery listed above. Finally, this review also highlights that more attention is needed on a practical and research level into the process of family recovery within a mental health context.

## Figures and Tables

**Table 1:** Initial Inclusion/Exclusion Criteria.

Inclusion Criteria	Exclusion Criteria
Qualitative research articles.	Quantitative papers, editorials, discussion papers, literature reviews/systematic reviews/meta-synthesis, meta-analysis.
English Language.	
Peer-reviewed.	
General adult mental health services.	Addiction, Intellectual Disabilities, Physical Health, Older Person Services–Dementia, Delirium, etc, child and adolescent services, dual diagnosis.
	Dissertations.
Articles focussed on Family members/carers’ interventions.	Article focussed on users of service.
Articles published within the past 10 years.	

**Table 2:** Comparative appraisal of included studies.

Authors/Year/ Geographical Location	Study Aim	Sample and Sample Size	Age Range	Setting	Methodological Approach	Theoretical Orientation
[49] United Kingdom	To ascertain the views of relatives on how to design a supported self-management intervention for relatives	Relatives–Parents/Partners (*n* = 23)	53–63 Years	Non-NHS Community Setting	N/S	N/S
[50] 2015 United Kingdom	To describe the development/content/structure of a pilot family psychoeducation programme	Families–Parents/Siblings (*n* = 10)	N/S	Low Security Forensic Mental Health Services	N/S	N/S
[51] 2014 United Kingdom	To present a qualitative analysis of the Kidstime programme	Children/Young People, Former Service Users (*n* = 15)	4–16 Years	Non-NHS Community Setting	N/S	N/S

**Table 3:** Critical appraisal tool: Results of the quality assessment for the qualitative studies (*n* = 3).

Study	Abstract/Title	Introduction/Aims	Data Collection	Sampling	Analysis	Ethics/Bias	Results	Generalisability	Implications	Total	* Grade
[49]	3	4	3	2	3	2	4	2	1	24	C
[51]	3	3	3	2	3	2	3	2	1	22	C
[50]	3	4	1	2	1	1	2	1	2	20	C

* Grading key: High quality (A), 30–36 points; medium quality (B), 24–29 points; low quality (C), 9–24 points.

**Table 4:** Qualitative synopsis of included studies.

Authors	Synopsis of Included Studies
[49] United Kingdom	Family interventions can help to improve outcomes for people with psychosis and their families by reducing hospital admissions and relapse rates. Interventions which reduce stress for families of persons experiencing psychosis should be readily available. This study seeks to elicit the views of families on the content development of a self-management toolkit for families. Qualitative methods were utilised using a sample of adult parents and partners (*n* = 23). The gender of participants was male (*n* = 11) and females (*n* = 12).
[50] United Kingdom	There is a dearth of family interventions within forensic mental health settings. This study reports on the development, content, implementation and evaluation of a psychoeducation programme for families with loved ones in forensic mental health settings. Qualitative methods were employed using a sample of adult parents and siblings (*n* = 10). The gender of participants was not documented in this study.
[51] United Kingdom	Children of parents with mental health challenges have a 30–50% chance of becoming seriously mentally unwell themselves. Kidstime is an interactive programme that attempts to address the needs of such children and adolescents. The present study seeks to evaluate Kidstime by exploring service user perspectives. Qualitative methods were used to achieve this using a sample of children, young people and former service users (*n* = 15). The gender of participants was not documented in this study.

**Table 5:** Themes and subthemes.

Themes	Sub-Themes
Family Recovery Initiatives	Recovery Toolkit
	Family Psychoeducation
	Kidstime
Benefits	Education
	Social Inclusion
	Facilitation of Discussion of Difficult Topics
Challenges	Hidden Interventions
	Practicalities
	Age Appropriate Interventions
Enablers for Recovery Interventions	Written Information
	Access
	Supports
	Decision Makers for Attending Interventions

## Data Availability

Not applicable.

## Appendix A. Detailed Inclusion/Exclusion Criteria Based on PICO

**PICO**	**Inclusion Criteria**	**Exclusion Criteria**
Population.	- Parents of adult children with mental health difficulties. - Parents of children with mental health difficulties. - Children of parents with mental health difficulties. - Adult children of parents with mental health difficulties. - Siblings of children with mental health difficulties. - Siblings of adults with mental health difficulties. - Spouses of persons with mental health difficulties. - Carers’ of adults with mental health difficulties. - Carers’ of children with mental health difficulties.	- Service users with mental health difficulties. - Service users with addictions. - Service users with a dual diagnosis. - Service users of physical health services. - Family members of loved ones in addiction services. - Family members of loved ones in physical health services. - Carers’ of service users of addiction or physical health services. - Family members/carers of loved ones with a dual diagnosis.
Intervention	- Any individual or group-based interaction, that includes clinician to family member, peer-to-peer, online synchronise, asynchronis, self-help, recovery education, recovery colleges that focus specifically on family members/carers. - Interventions that focus on family/carer recovery in their own right. - Interventions that can focus on both service users’ and family members’/carers’ recovery.	- Interventions that focus on service users’ recovery.
Comparison	- Treatment as usual.	- Treatment that is inclusive of family members/carers’ recovery - Treatment of service users.
Outcomes	- The effectiveness of such an intervention on family members/carers’ own recovery.	- Process evaluation of such interventions. - Service user recovery outcomes.

## Appendix B. Detailed PICO Explanation for this Systematic Review

**Population**	Studies were included if they reported about families with loved ones attending mental health services. These loved ones must have a diagnosis of any mental health condition described within the following diagnostic manuals: International Classification of Diseases (ICD-11) and the Diagnostic and Statistical Manual for Mental Disorders (DSM-5). However, exclusions applied in terms of disorders relating to cognitive decline such as dementia or delirium and those specifically relating to intellectual disabilities. These exclusions were applied as such disorders are also mentioned within these diagnostic manuals.
**Intervention**	Papers were included if they discussed recovery interventions that focused on family members in their own right. The definition of recovery interventions that were incorporated into the search strategy related to any intervention (individual or group-based interaction that includes clinician to family member, peer-to-peer, online synchronous, asynchronous, self-help, recovery education, recovery colleges) that increases or decreases the wellbeing of family members or carers of those with a mental health challenge.
**Comparison**	The comparison made for this systematic review was treatment as usual.
**Outcomes**	The outcome which the reviewers seek to capture relates to the efficacy of such interventions for a family member/carer recovery.

## Appendix C. Detailed Search Strategy Family Recovery Interventions: A Systematic Review

Question: What recovery interventions exist for families of loved ones attending mental health services that focus on the family member in their own right.

P—Families with loved ones attending mental health services

I—recovery interventions that focus on family in their own right

C—Treatment as usual

O—To understand the efficacy of such interventions

### Appendix C.1. Search String

(Family OR Supporter* OR Spouse* OR Partner* OR “Significant other” OR Caregiver* OR Relative* OR Sibling* OR Parent*) AND (Adult*) AND (Mental OR Psychiatric or “mental illness” or “psychiatric disorder” OR “mental ill health”) AND (intervention OR “group work” OR therapy* or education OR support) AND (recovery OR wellbeing OR “self-care” OR “burden” OR “quality of life”)

Mental health–any diagnosed mental health condition as per ICD-11, DSM5.

Intervention–any individual or group-based interaction, that includes clinician to family member, peer-to-peer, online synchronise, asynchronis (not live), self-help, recovery education, recovery colleges that focus specifically on family members/carers’.

Recovery/wellbeing–Increasing/decreasing the wellbeing of family members/carer.

### Appendix C.2. Database

Multi-search (CINAHL, JSTOR, OVID SP, PsycARTICLES, PsycINFO, PubMed, Science Direct, Web of Science, Wiley Online Library) (Michael) EBSCHO host (Kerry), other resources (i.e., Google Scholar, ResearchGate).

### Appendix C.3. Inclusion/Exclusion

**Inclusion**	**Exclusion**
Qualitative research articles	Quantitative papers, editorials, discussion papers, literature reviews/systematic reviews/meta-synthesis, meta-analysis
English Language	
Peer reviewed	
General adult mental health services	Addiction, Intellectual Disabilities, Physical Health, Older Person Services–Dementia, Delirium, etc, child and adolescent services, dual diagnosis
	Dissertations
Articles focussed on Family members/carers’ interventions	Article focussed on users of service
Articles published within the past 10 years	

### Appendix C.4. Systematic Process

Round 1—Review titles from search results–anything to do with family, recovery is saved to round 1 folder–folder divided into those found in databases and other source documents

Round 2—Saved article abstracts are read. Duplicates removed. Inclusion/Exclusion applied to remove any documents not relating to the research question

Round 3—Full articles reviewed–Inclusion/Exclusion criteria fully implemented
